# Complete Genome Characterization of the 2017 Dengue Outbreak in Xishuangbanna, a Border City of China, Burma and Laos

**DOI:** 10.3389/fcimb.2018.00148

**Published:** 2018-05-08

**Authors:** Songjiao Wen, Dehong Ma, Yao Lin, Lihua Li, Shan Hong, Xiaoman Li, Xiaodan Wang, Juemin Xi, Lijuan Qiu, Yue Pan, Junying Chen, Xiyun Shan, Qiangming Sun

**Affiliations:** ^1^Institute of Medical Biology, Peking Union Medical College, Chinese Academy of Medical Sciences, Kunming, China; ^2^Yunnan Key Laboratory of Vaccine Research and Development on Severe Infectious Diseases, Kunming, China; ^3^Yunnan Key Laboratory of Vector-borne Infectious Disease, Kunming, China; ^4^Xishuangbanna Dai Autonomous Prefecture People's Hospital, Xishuangbanna, China; ^5^School of Basic Medicine, Kunming Medical University, Kunming, China; ^6^Institute of Pediatric Disease Research, The Affiliated Children's Hospital of Kunming Medical University, Kunming, China

**Keywords:** dengue outbreak, complete genome, phylogenetic trees, RNA secondary structure, protein secondary structure

## Abstract

A dengue outbreak abruptly occurred at the border of China, Myanmar, and Laos in June 2017. By November 3rd 2017, 1184 infected individuals were confirmed as NS1-positivein Xishuangbanna, a city located at the border. To verify the causative agent, complete genome information was obtained through PCR and sequencing based on the viral RNAs extracted from patient samples. Phylogenetic trees were constructed by the maximum likelihood method (MEGA 6.0). Nucleotide and amino acid substitutions were analyzed by BioEdit, followed by RNA secondary structure prediction of untranslated regions (UTRs) and protein secondary structure prediction in coding sequences (CDSs). Strains YN2, YN17741, and YN176272 were isolated from local residents. Stains MY21 and MY22 were isolated from Burmese travelers. The complete genome sequences of the five isolates were 10,735 nucleotides in length. Phylogenetic analysis classified all five isolates as genotype I of DENV-1, while isolates of local residents and Burmese travelers belonged to different branches. The three locally isolates were most similar to the Dongguan strain in 2011, and the other two isolates from Burmese travelers were most similar to the Laos strain in 2008. Twenty-four amino acid substitutions were important in eight evolutionary tree branches. Comparison with DENV-1SS revealed 658 base substitutions in the local isolates, except for two mutations exclusive to YN17741, resulting in 87 synonymous mutations. Compared with the local isolates, 52 amino acid mutations occurred in the CDS of two isolates from Burmese travelers. Comparing MY21 with MY22, 17 amino acid mutations were observed, all these mutations occurred in the CDS of non-structured proteins (two in NS1, 10 in NS2, two in NS3, three in NS5). Secondary structure prediction revealed 46 changes in the potential nucleotide and protein binding sites of the CDSs in local isolates. RNA secondary structure prediction also showed base changes in the 3′UTR of local isolates, leading to two significant changes in the RNA secondary structure. To our knowledge, this study is the first complete genome analysis of isolates from the 2017 dengue outbreak that occurred at the border areas of China, Burma, and Laos.

## Introduction

Dengue virus (DENV) is a member of the family *Flaviviridae* and genus *Flavivirus*, which has four closely related serotypes (DENV-1 to DENV-4) (Anez et al., 2017). Among these serotypes, DENV-1 has five distinct genotypes (I–V) (Sasmono et al., 2015). DENV is a single-stranded positive-sense RNA virus approximately 11 kb in length, comprising an open reading frame (ORF) and 5′ and 3′untranslated regions (UTRs). Translation of the ORF encodes three structural proteins (capsid [C], membrane [PrM/M], and envelope [E]) and seven non-structural proteins (NS1, NS2A, NS2B, NS3, NS4A, NS4B, and NS5) (Nukui et al., 2006; Parameswaran et al., 2017).

Dengue is a highly prevalent disease spread by the mosquito species *Aedes albopictus* and *Ae. aegypti* (Lustig et al., 2017). This virus is endemic to more than 100 countries worldwide, especially in cities and semi-urban areas of tropical and subtropical countries (Imrie et al., 2010; Anez et al., 2017). A recent study estimated that 390 million cases of dengue infection occur worldwide each year, representing a 30-fold increase in the past 50 years (Anez et al., 2017). Dengue virus has become a worldwide public problem (Santos-Sanz et al., 2014). The World Health Organization (WHO) classified dengue disease as dengue fever (DF), dengue hemorrhagic fever (DHF), and dengue shock syndrome (DSS) in 1997 (Narvaez et al., 2011). However, in 2008, the WHO proposed a new classification system, classifying dengue into dengue without warning signs (DWWS), dengue with warning signs (DWS), and severe dengue (SD) (Lima et al., 2013).

The first dengue outbreak in China was reported in Foshan, Guangdong Province, in 1978. Since then, dengue epidemics have been reported in Guangdong, Jiangsu, Hainan, Fujian, Guangxi, Zhejiang, and Henan (Huang et al., 2014; Liu et al., 2014; Xiong and Chen, 2014; Chen and Liu, 2015; Ren et al., 2015). In 2008, an unexpected dengue outbreak occurred in Ruili, a city on the border of China and Burma (Zhang et al., 2013). In 2013 and 2015, large-scale dengue outbreaks occurred in Xishuangbanna, Yunnan Province, a Chinese city on the border of China, Burma, and Laos. In 2014 and 2016, sporadic cases of dengue fever were also reported. In June 2017, a dengue outbreak developed abruptly at the borders of China, Burma, and Laos. By 03 November 2017, 1,184 infected individuals in Xishuangbanna had been confirmed as dengue NS1-positive.

## Materials and methods

### Ethics statement

Each participant was informed of the purpose of the study. Written informed consent was obtained from all participants. The study protocol was approved by the Institutional Ethics Committee (Institute of Medical Biology, Chinese Academy of Medical Sciences, and Peking Union Medical College). The study was conducted in accordance with the Declaration of Helsinki for Human Research of 1974 (last modified in 2000).

### Dengue virus RNA extraction and identification

Serum samples were collected from DENV NS1-positive patients at Xishuangbanna Dai Autonomous Prefecture People's Hospital. The serum samples were separated from the collected blood, followed by viral RNA extraction. Viral RNA was extracted from 140 μl of dengue virus-infected serum using the QIAamp viral RNA mini kit (Qiagen, Hilden, Germany; No. 52906). The RNA was eluted in 60 μl of nuclease-free water and stored at −80°C. Viral RNA was reverse transcribed into cDNA using the PrimeScript™ II 1st Strand cDNA Synthesis Kit (Takara Bio, Shiga, Japan; No. 6210A). The cDNA was amplified by using dengue universal, 1, 2, 3, 4-type primers (Supplementary Table 1) for specific identification. The reaction conditions in each 25-μl volume were denaturation at 94°C for 5 min, followed by 35 cycles of denaturation at 94°C for 30 s, annealing at 55°C for 30 s, and elongation at 72°C for 30 s; with a final elongation step at 72°C for 7 min. The PCR products were then subjected to gel electrophoresis.

### Primer design

A total of 21 synthetic oligonucleotide primer pairs (Supplementary Table 2) were designed to amplify overlapping fragments with sizes between 200 and 900 nucleotides (nt) spanning the entire viral genome of DENV-1. All primers were selected online from Primer-BLAST at the NCBI website, based on the strain WestPac (GenBank accession no. U88535.1). All primers were synthesized and purified by Shuo GE Biotechnology Co., Ltd. (Kunming, China).

### Genome synthesis for amplification and sequencing

PCR was performed with the following protocol (50 μl volume): denaturation at 98°C for 2 min, followed by 40 cycles of denaturation at 98°C for 10 s, annealing at 56°C for 10 s, and elongation at 72°C for 30 s; with a final elongation step at 72°C for 1 min. The PCR products were confirmed by agarose gel electrophoresis and sequenced at Shuo GE Biotechnology Co. Ltd. Forward and reverse sequencing were done.

### Analysis of molecular characteristics

A total of 21 nucleotide sequences were assembled using BioEdit7.1.3 (http://www.mbio.ncsu.edu/bioedit/bioedit.html). Complete genome sequences of five DENV-1 isolates were submitted to the NCBI GenBank database with accession numbers of MF681692 (for YN2), MF683116 (YN1774), MF681693 (YN176272), MG679800 (MY21), and MG679801 (MY22). Nucleotide sequences and translated amino acid sequences were analyzed by BioEdit7.1.3. The secondary structure of isolates and reference strains CDS and UTR was predicted with the Predict Protein server (https://www.predictprotein.org/) and RNA fold web server (http://rna.tbi.univie.ac.at/cgi-bin/RNAWebSuite/RNAfold.cgi), respectively.

### Phylogenetic analysis

The sequences were assembled using BioEdit 7.1.3. Phylogenetic analysis based on the whole genome genes was conducted using the Molecular Evolutionary Genetics Analysis (MEGA) software version 6.0 (Maximum Likelihood method (ML), Bootstrap1000, Jones-Taylor-Thornton (JTT) model). The reference viral sequences used to construct the distinct phylogenetic branches were collected from the GenBank sequence database under the following country accession numbers: China (JQ048541, KP7752, KU365900, DQ193572, FJ176780, AB608788, KU094071, and KT827374), Singapore (GU370049, KJ806945, M87512, JN544411, and GQ357692), Malaysia (KX452068 and KX452065), South Korea (KP406802), Indonesia (KC762646), Japan (AB178040), Thailand (HM469966 and AF180817), Myanmar (AY726554 and AY726553), Laos (KC172829, KC172835, and KC172834), Cambodia (HQ624984), Viet Nam (JQ045660), Hawaii (EU848545, DQ672564, and DQ672562), Western Pacific (U88535), South Pacific (JQ915076), Brazil (AF226685), Haiti (KT279761), USA (FJ390379), India (KF289072), and standard strains (M29095, M93130, and AF326573).

## Results

### Geographic analysis of the outbreak

Xishuangbanna has become the central area of dengue fever epidemics occurring at the borders of China, Myanmar, and Laos. The latest was an abrupt onset outbreak that began in June 2017. As of November 3rd 2017, 1,184 infected individuals had been confirmed as dengue NS1-positive as of November 3rd in Xishuangbanna, Yunnan, China. These cases included 71 cases involving non-residents of Xishuangbanna and 1,113 local cases.

### Base sequence analysis of the complete genome sequences of five DENV isolates

The complete genome sequences of five isolates were obtained by amplifying 21 overlapping fragments. Two of the five complete genome sequences were obtained from travelers from Burma. The remaining three genome sequences were obtained from local residents. Sequencing and clustering, All isolates were 10,735 nt in length. The 5′ and3′UTRs of the five isolates were 94 and 465 nt in length, respectively. The ORF of all five isolates was located between nt 95 and 10270, and encoded 3,392 amino acids.

### Phylogenetic analysis

**Figure 1:**
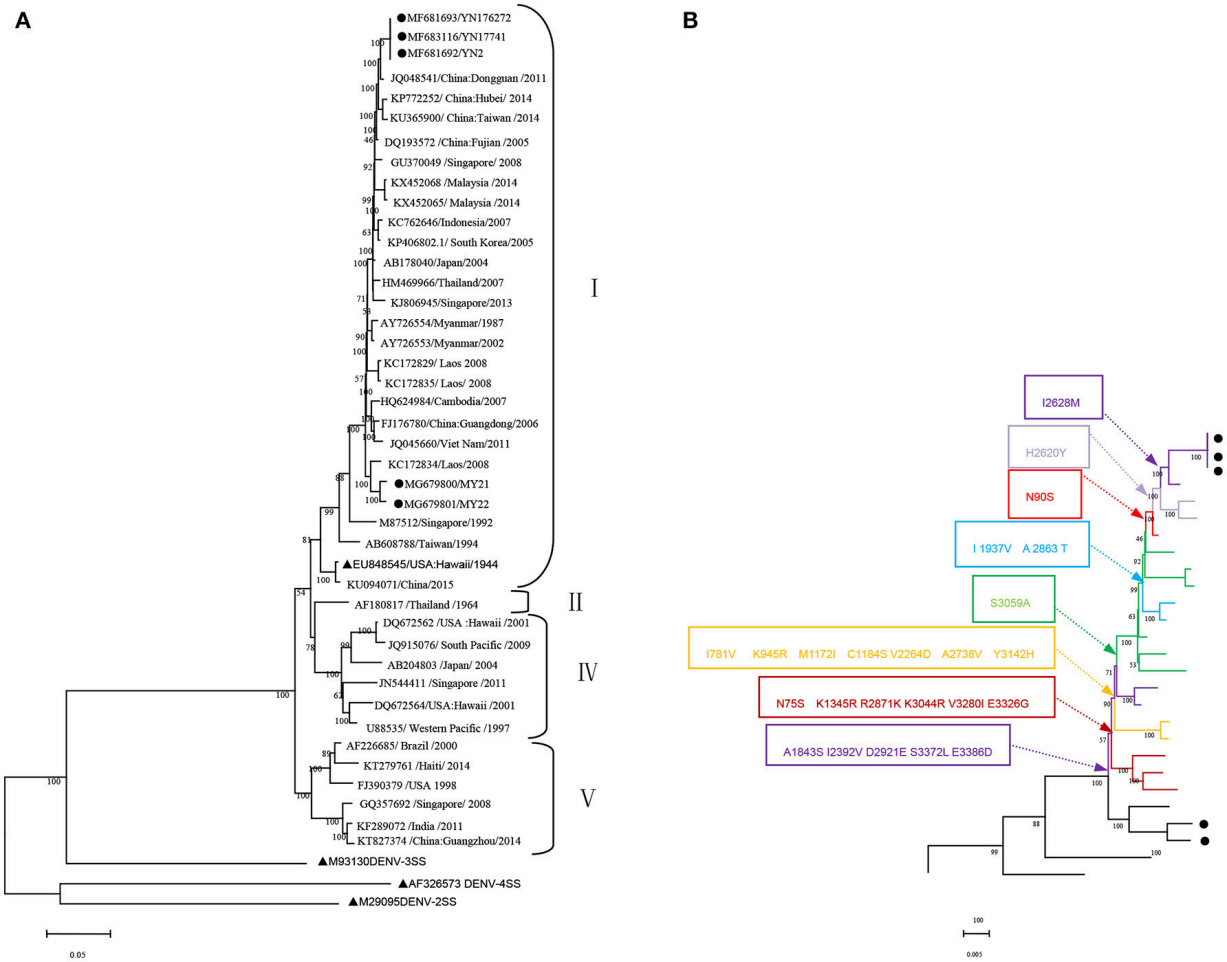
Phylogenetic tree data. **(A)** Phylogenetic tree of DENV-1 epidemic strains in Xishuangbanna, Yunnan, China, in 2017. The phylogenetic tree was constructed with the Maximum Likelihood method with 1,000 bootstrap values. • isolates from Xishuangbanna, Yunnan, China; ▴ standard strains of DENV1-4 serotypes. **(B)** Relationship between evolutionary tree branching and amino acid mutations in genotype I of DENV-1.

The whole genome of the five isolates was aligned and compared with 40 sequences from standard strains of four serotypes and some typical DENV-1 strains of various geographical origins obtained from the NCBI GenBank database. The phylogenetic analysis was constructed with the ML method. The five isolates belonged to DENV-1 and were further classified as genotype. Three local isolates were located in one cluster of the ML tree, with the closest relationship to the Dongguan strain of China (2011, JQ048541). Additionally, three isolates were also closely related to the following strains: China (Hubei 2014 KP772252, Taiwan 2014 KU365900, and Fujian 2005 DQ193572), Singapore (2008, GU370049), and Malaysia (2014 KX452068 and KX452065). However, two isolates obtained from Burmese travelers were located in another cluster of the ML tree and were most similar to the Laos strain in 2008 (KC172834). DENV-3 (M93130), DENV-4 (AF326573), and DENV-2 (M29095) were outgroups (Figure 1A).

**Figure 2:**
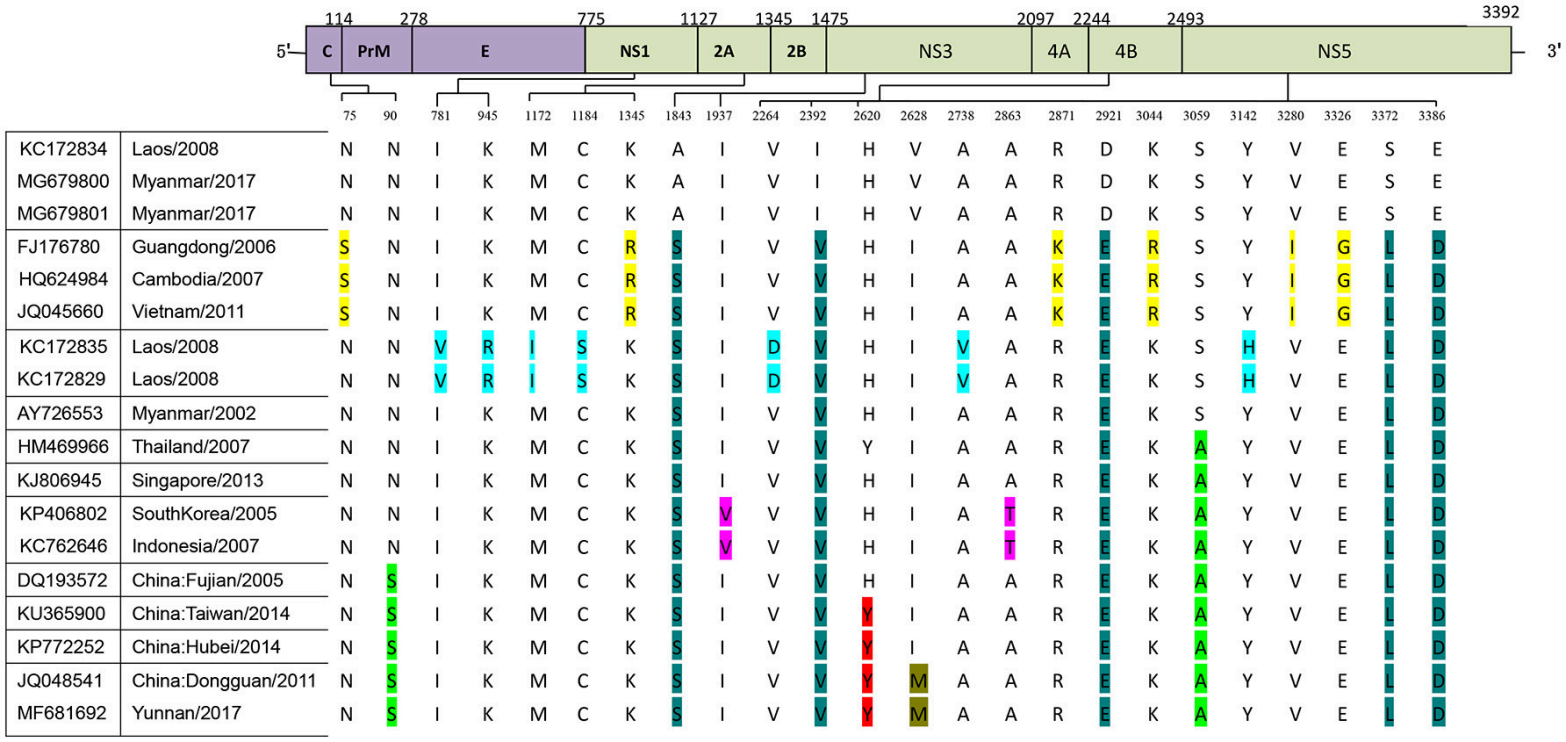
Identification of variations among genotype I of DENV-1. Variations are presented in accordance with their location in the viral genome. The various colors indicate the different variations described in Figure 1B.

We further analyzed the relationship between the evolutionary tree branching and amino acid substitution of genotype ? of DENV-1. A total of 24 amino acid substitutions were detected that had important roles in eight evolutionary treebranches. Particularly, A1843S, I2392V, D2921E, S3372L, and E3386D resulted in branch 1; N75S, K1345R, R2871K, K3044R, V3280I, and E3326G resulted in branch 2; I781V, K945R, M1172I, C1184S, V2264D, A2738V, and Y3142H resulted in branch 3; and S3059A, I1937V/A2863T, N90S, H2620Y, and I2628M resulted in branches 4–8, respectively (Figures 1B, 2).

### Base substitution and amino acid mutation analysis of coding DNA sequences (CDSS) from the isolated strains

Base substitution and amino acid mutation analyses were performed by comparison with the DENV-1 standard strain (DENV-1SS) (Hawaii, 1944, NO. EU848545). The results revealed 187 base substitutions in the structural protein (C/PrM/E) in three local isolates, of which 155 substitutions were non-synonymous. These were 471 base substitutionsin non-structural protein regions in the three local isolates, leading to 416 non-synonymous mutations (Supplementary Figure 1). The genome sequence of the three local isolates was almost the same, except for nt 3786(C → T) and 10630(A → G) in isolate YN17741. All the isolates harbored a base deletion (T) at nt 79 of the 5′UTR, and a few base substitutions occurred in the 3′UTRs in all the isolates.

**Figure 3:**
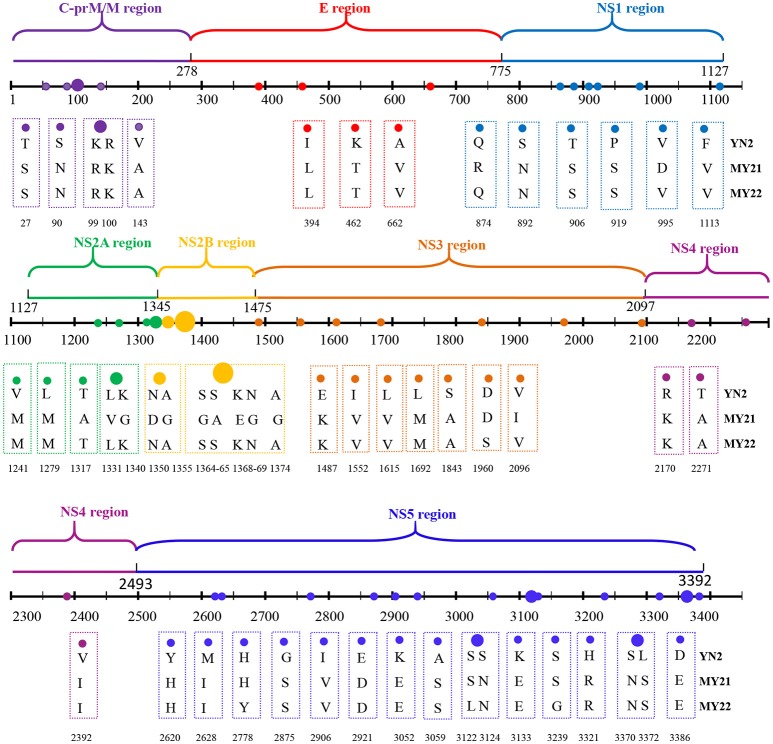
Amino acid mutations between CDS of locally isolated strains and two strains isolated from Myanmar (2017).

The two isolates obtained from Burmese travelers were different from the three local isolates. Compared with local isolates, a total of 52 amino acid mutations occurred in the CDS of the two isolates (MY21 and 22) obtained from Burmese travelers. There were also differences between MY21 and 22. Comparison of MY21with MY22 revealed 17 amino acid mutations, which all occurred in CDS of non-structural proteins. Of these, two amino acid mutations (R874Q, D995V) occurred in the non-structural protein NS1, three amino acid mutations (A1317T, V1331L, G1340K) occurred in the non-structural protein NS2A, seven amino acid mutations (D1350N, G1355A, G1364S, A1365S, E1368K, G1369N, G1374A) occurred in the non-structural protein NS2B, two amino acid mutations (D1960S, I2096V) occurred in the non-structural protein NS3, and three amino acid mutations (H2778Y, S3122L, S3239G) occurred in the non-structural protein NS5 (Figure 3).

**Figure 4:**
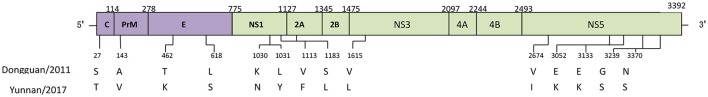
Amino acid mutations between CDS of Dongguan strain (2011) and Yunnan strains (2017).

Compared to the closest Dongguan strain (China 2011, JQ048541), a total of 14 amino acid mutations occurred in the CDS of the local isolates (Figure 4). Three mutations leading to changes in amino acid polarity occurred in non-structural protein regions. At amino acid 1031in L1031Y, the amino acid changed from a non-polar to a polar residue, while the change was from polar to non-polar residues in positions S1183L and V1615L).

### Prediction of the RNA secondary structure based on the untranslated regions of the standard and local isolated strains

All sequences wereconserved in the UTRs. Compared to the standard strain, only one base deletion occurred, and thus the predicted RNA structure of the 5′UTR was only marginally changed (Supplementary Figures 2A,B). Comparison with the 3′UTR of the standard strains revealed that all local isolates had eight substitutions (nt 10273 [A → G], nt 10276 [T → C], nt 10286 [C → T], nt 10293 [C → T], nt 10408 [G → A], nt 10467 [C → T], nt 10536 [A → G], nt 10621 [C → T]) and 3 inserts (nt 10455 [G], nt 10479 [A], and nt 10730 [C]). In addition to these mutations, an additional mutation at nt 10630 [A → G] was observed in YN17741. RNA secondary structure prediction results showed that some base changes in the 3′UTR of the local isolates lead to two significant changes in the RNA secondary structure. In contrast to the long interior loop in the standard strain, a 47 bp stem downstream of nt 10277 was formed in the isolates. Long stem and loop structures were formed before nt 10734 in the standard strain, while the stem and loops were shorter in the isolates before nt 10719 (Supplementary Figures 2C,D).

### Potential secondary structure of the CDS regions

The potential secondary structures of the CDS regions of the local isolates were predicted and compared with that of the standard strain. In structural proteins C/prM/E, there were 13 changes inputative nucleotide/protein binding sites among the 775 amino acids. Among these, six putative protein-binding sites (sites 8, 68, 87, 161, 335, and 436) were detected only in the isolates. However, five potential protein-binding sites (sites 1, 7, 21, 86, and 429) and two nt-binding sites (sites 6 and 7) were lost compared to the standard strain. Approximately eight distinct changes appeared in the helix and strand regions. Two distinct changes were found in the exposed and buried regions and the disordered regions. The helical transmembrane regions in the local isolates were highly consistent with those in the standard strain (Supplementary Figure 3). In the non-structural protein NS1, comparison with the standard strain revealed only one potential nucleotidesite (site 118) and protein binding site (site 99) in the isolates. Approximately four distinct changes were found in the helix and strand regions (Supplementary Figure 3B). In the non-structural protein NS2A, three changes were observed in putative protein binding sites among the 218 amino acids. Among these changes, four potential protein-binding sites were increased, and two other sites (118 and 180) were lost in the isolated strains (Supplementary Figure 3C). In the non-structural protein NS2B, comparison with reference strain revealed only one change (site 49) in the putative protein binding sites of the isolates (Supplementary Figure 3D). In the non-structural protein NS3, 15 changes were observed in the putative nucleotide and protein binding sites among the 619 amino acids. Among these changes, five potential protein-binding sites (sites 132, 134, 143, 338, and 471) were increased in the isolates, while 10 putative protein-binding sites (sites 107, 161, 172, 304, 461, 481, 483, 531, 539, and 593) were lost compared with the standard strain (Supplementary Figure 3E). In the non-structural protein NS4A, only two distinct changes were found in the exposed and buried regions and disordered regions (Supplementary Figure 3F). In the non-structural protein NS4B, the isolates lost potential nucleotide (site 249) and protein binding sites (site 243) compared to the standard strain (Supplementary Figure 3G). In the non-structural protein NS5, 11 changes were detected in putative nucleotide and protein binding sites among the 899 amino acids. Four putative protein-binding sites (sites 51, 289, 558, and 691) and two nucleotide-binding sites (sites 598, 853) were increased in the isolated strains, while five putative protein-binding sites (sites 350, 465, 499, 502, and 895) were lost. In addition, three distinct changes were found in the disordered regions (Supplementary Figure 3H).

## Discussion

Yunnan Province is located on the southwest border of China. The northwest corner is closely attached to the Tibet autonomous region. The west borders Myanmar and the south is connected with Laos and Vietnam. Yunnan is located in the vast expanse of the Asian continent, south of the Indian Ocean and the Pacific Ocean. The region is influenced by monsoons that originate to the southeast and southwest, and also by the Tibetan plateau area. These climatic influences have produced a complex and diverse natural geographical environment. The region is a popular touristic destination. These and other factors have made this area important for the prevention and control of dengue fever in Asia.

A 2006 study revealed the existence of DENV infection in the Dushan and Xingyi areas of the Yunnan-Guizhou Plateau, China (Liu et al., 2006). In 2008, 48 imported cases and one native case were reported in Yunnan Ruili. This was the first report of a dengue outbreak in Yunnan. Of the 49 confirmed cases, 18 involved people from Mujie City, Myanmar, 30 cases involved Chinese residents who had returned to Myanmar from elsewhere, and one native case. This outbreak was caused by DENV-1 and DENV-3 (Zhang et al., 2013). A DENV-2 isolate was obtained from a patient with fever who returned from Laos in 2009 (Guo et al., 2013). The first aboriginal outbreak occurred in Xishuangbanna Dai Autonomous Prefecture, Yunnan Province, China, in 2013. This outbreak was caused by DENV-3 and 1,331 cases were confirmed. A mixed outbreak of DENV-1 and DENV-2 appeared in Ruili in Dehong prefecture, with 246 dengue fever cases recorded (Zhang et al., 2014; Guo et al., 2015; Wang et al., 2017). From June to December of 2014, a total of 292 cases of dengue fever were diagnosed in Ruili City. These included 139 local cases and 153 imported cases caused by DENV-1, DENV-2, and DENV-4. In 2015, 240 DENV-1 cases were reported in Lincang, and 1,067 DENV-2 cases were reported in Xishuangbanna (Guo et al., 2016; Zhao et al., 2016). In 2016, 25 isolates were obtained from the sera of dengue fever patients. These included 15 local cases and 10 imported cases from Burma. These cases were caused by DENV-1, DENV-2, DENV-3, and DENV-4. A dengue fever outbreak caused by DENV-1occurred in Xishuangbanna, Yunnan Province, in 2017. This was the first known outbreak due to DENV-1in this area. According to government statistics, as of 03 November 2017, a total of 1,184 infected cases have been verified, including 71 imported cases and 1,113 local cases. Interestingly, large-scale dengue outbreak shave occurred in Xishuangbanna every 2 years since 2013, involving different serum types of DENV.

In the present study, viral RNA was extracted from the sera of patients from China and Myanmar, followed by amplification of overlapping fragments to obtain five complete genomes (YN2, YN17741, YN176272, MY21, and MY22). Two isolates (MY21 and MY22) were obtained from Burmese travelers. The five isolates were 10,735 bp in length. YN2 and YN176272 displayed the same genome sequence. Compared with YN2, YN17741 possessed has two base changes at nt 3786 (C → T) and nt 10630 (A → G). These base changes were non-synonymous. However, the two isolates from Burmese travelers were different from three isolated strains from local residents. Compared with locally isolated strains, a total of 52 amino acid mutations occurred in the CDS of the two isolates from Burmese travelers. These two isolates differed from each other. Comparison of MY21 and MY22 revealed 17 amino acid mutations. All occurred in CDS of non-structured proteins. These results indicate that the 2017 dengue outbreak in Xishuangbanna was not caused by the imported DENV-1, but rather was a local outbreak. The 2017 dengue outbreak in the border areas around Xishuangbanna was not caused by only one DENV-1 source strain, but belong to a single genotype.

The 5′ and 3′ UTRs of dengue virus play an important role in the genome replication of RNA (Wei et al., 2008; Vashist et al., 2009). According to different conservative levels, the 3′ UTR is divided into three regions: (1) a variable area directly following the ORF; (2) a highly conserved cyclization sequence motif (CS1) and stable stem-loop structure; and (3) the region between the variable and 3-terminal regions, which have modest conservative levels and contain several hairpin motifs (Bryant et al., 2005). The 3′UTR is dispensable for the replication of dengue type 1 virus *in vitro* (Tajima et al., 2006). The variable area in the 3' UTR of Japanese encephalitis virus (JEV) reportedly did not affect its growth *in vitro* or its pathogenicity in mice (Kato et al., 2012). However, a previous report showed that the variable area of DENV-2 can enhance viral replication in baby hamster kidney cells, although this area is dispensable in mosquito cells (Alvarez et al., 2005). Another study showed that VR mutations of DENV-1 might not affect the translation process (Tajima et al., 2007). In the present study, mutations were observed in the 3′UTRs of the isolates compared to those of the reference strains. Nucleotide changes in 3′UTRs of the isolates lead to two significant changes in the RNA secondary structure. In contrast to the long interior loop in the standard strain, a 47-bp stem downstream of nt 10277 was formed in the isolated strains. Long stem and loop structures were formed before nt 10734 in the standard strain, while the stem and loops were shorter in the isolated strains before nt 10719, especially in the variable area. The effect of these changes on dengue type 1 replication needs further clarification.

In recent years, several studies reported that the mutation of amino acids in some special sites has a significant impact on the virulence, replication, and infectivity of flaviviruses. An alanine-to-valine amino acid substitution at residue 188 of the NS1 protein promotes the infectivity of Zika virus (ZIKV) Asian genotype in mosquitoes (Delatorre et al., 2017; Liu et al., 2017). A single mutation (S139N) in the PrM protein of Zika virus promotes fetal microcephaly (Yuan et al., 2017). The single mutation G66A in the NS2A gene of Japanese encephalitis-live vaccine virus reduces neurovirulence and neuroinvasiveness in mice(Ye et al., 2012). The mutation of putative N-glycosylation sites (Asn-58 and Asn-62) affects the biological function of DENV NS4B in the viral replication complex (Naik and Wu, 2015). A DENV-2 vaccine strain (PDK53) has three mutations that are critical for the attenuated state of PDK53 (Butrapet et al., 2000). The present results indicate that the region of the viral structural protein is relatively conserved, with less amino acid substitutions, with mutations being more prevalent in the non-structural protein regions in genotype I of DENV-1. Based on phylogenetic analysis, we identified a total of 24 amino acid substitutions that play an important role in evolutionary tree branching. These substitutions included two important amino acid substitutions (N90S and S3059A) in Chinese strains (DQ193572, KU365900, KP772252, JQ048541, and MF681692), and an amino acid substitution (I2628M) in the local isolated strains and the Dongguan strain of China (JQ048541). The effects of these amino acid substitutions on virulence, replication, infection, and other functions of the virus are unclear.

In 2017, there have been extensive outbreaks of dengue fever in many areas including Yunnan, Guangdong, Zhejiang, Shandong, Taiwan, Xianggang, Macao, and other areas (Chen et al., 2017). Xishuangbanna is geographically near Myanmar, Laos, and Vietnam. The present study is the first report on the complete genome characterization of the 2017 Xishuangbanna DENV outbreak. The present study should serve as a reference to follow-up studies of DENV outbreaks in Southeast Asia in 2017. The findings are a reference for future research on the virulence, replication, infection, pathogenicity, and vaccine development of DENV.

## Author contributions

XS and QS contributed to the design of the study. SW and DM were involved in data acquisition and analysis. YL, LL, SH, and XL provided software technology support. XW, JX, YP, LQ, and JC provided experiment technology support. All the authors agree to the final version of the submission.

### Conflict of interest statement

The authors declare that the research was conducted in the absence of any commercial or financial relationships that could be construed as a potential conflict of interest.
