# Supplementary material for: Complete Genome Characterization of the 2017 Dengue Outbreak in Xishuangbanna, a Border City of China, Burma and Laos
Source: Front Cell Infect Microbiol. 2018 May 8;8:148. doi: 10.3389/fcimb.2018.00148 (PMC5951998; doi:10.3389/fcimb.2018.00148)
Supplement: Supplementary file 1 [file Table_1.PDF]

Primer name	Forward primer	Reverse primer
DENV universal	TCAATATGCTGAAACGCGCGAGAAACCG	TTGCACCAACAGTCAATGTCTTCAGGTTC
DENV-1	TCAATATGCTGAAACGCGCGAGAAACCG	CGTCTCAGTGATCCGGGGG
DENV-2	TCAATATGCTGAAACGCGCGAGAAACCG	CGCCACAAGGGCCATGAACAG
DENV-3	TCAATATGCTGAAACGCGCGAGAAACCG	TAACATCATCATGAGACAGAGC
DENV-4	TCAATATGCTGAAACGCGCGAGAAACCG	CTCTGTTGTCTTAAACAAGAGA

Supplementary Table1. Primers for identification of dengue virus and its type.
