# Supplementary material for: Complete Genome Characterization of the 2017 Dengue Outbreak in Xishuangbanna, a Border City of China, Burma and Laos
Source: Front Cell Infect Microbiol. 2018 May 8;8:148. doi: 10.3389/fcimb.2018.00148 (PMC5951998; doi:10.3389/fcimb.2018.00148)
Supplement: Supplementary file 2 [file Table_2.pdf]

Primer name	Forward primer	Reverse primer
1	AGTTGTTAGTCTACGTGGACCG	GAGAACACGTCCCATAGGTCAC
2	ATGGACTTGGGAGAGTTATGTG	TCTCCTTGTGTTGGACATCTTG
3	CTGCGTCACCACCATGGCAAAA	TCCTGTTTCTTTGCATGAGCTG
4	TTTAATGAGATGGTGCTATTGA	CCTATGCTGCTTCCTCTCTT
5	AGAACCACCTTTTGGTGAGAGC	TTGGCATCTCCTACAACCAC
6	GTGTGGAATTCGATCAGCCACG	TTCACACAAGTCAAAATCCAGT
7	AGCCTCCTTCATAGAAGTCAAA	TACAGCGAACATTGGTCTCATT
8	TCTTATAATGGGACAACTGACA	TCCAATGATAAATCGGCTGAGC
9	TGATGTGCCGTTGGCCGGGCCA	CAACAGCTATCACCTGCACT
10	GGACTTGATCTCATATGGAGGA	CTAGCTGGATCGGTAAAATGTG
11	GTTGACCTCATGTGCCACGCTA	ATTTAAAGGCTGTCCCATATAA
12	TGCTGCCCAGAGGAGAGGAAGA	AGCTATCAAAGCTAGTAGCATC
13	ACATTTGACGTTAAGAGCCCAG	AACTCCTATGTCCATCTTCGAT
15	TTTCAGGGGAAGTTATCTAGCA	CACACTTGGCATGTAAGGATTT
16	GGCGACCTATGGATGGAACC	ATCCTCTGGCCAATGATATCTA
17	AGACATGTGGCAGTGGAACCAG	CCGGCTGTATCGTCTGCATA
18	GTTTAGTAGAGAGAATTCACTC	GGCACTTGCTGCCAATCATTCC
19	CTTAACAGCTCTGAATGACATG	TGTGTTGACTTACCAGAGTGCT
20	CACCTGGGCCACCAACATACAA	CTCTAACCACTAGTCTGCTACC
21	GCTCCATCGTGGGGATGTAAAA	GAACCTGGTTGATTCAACAGCACCA

Supplementary Table 2. 21 pairs of primers for amplifying whole genome of dengue type 1
