# Supplementary material for: Complete Genome Characterization of the 2017 Dengue Outbreak in Xishuangbanna, a Border City of China, Burma and Laos
Source: Front Cell Infect Microbiol. 2018 May 8;8:148. doi: 10.3389/fcimb.2018.00148 (PMC5951998; doi:10.3389/fcimb.2018.00148)
Supplement: Supplementary file 3 [file Image_1.PDF]

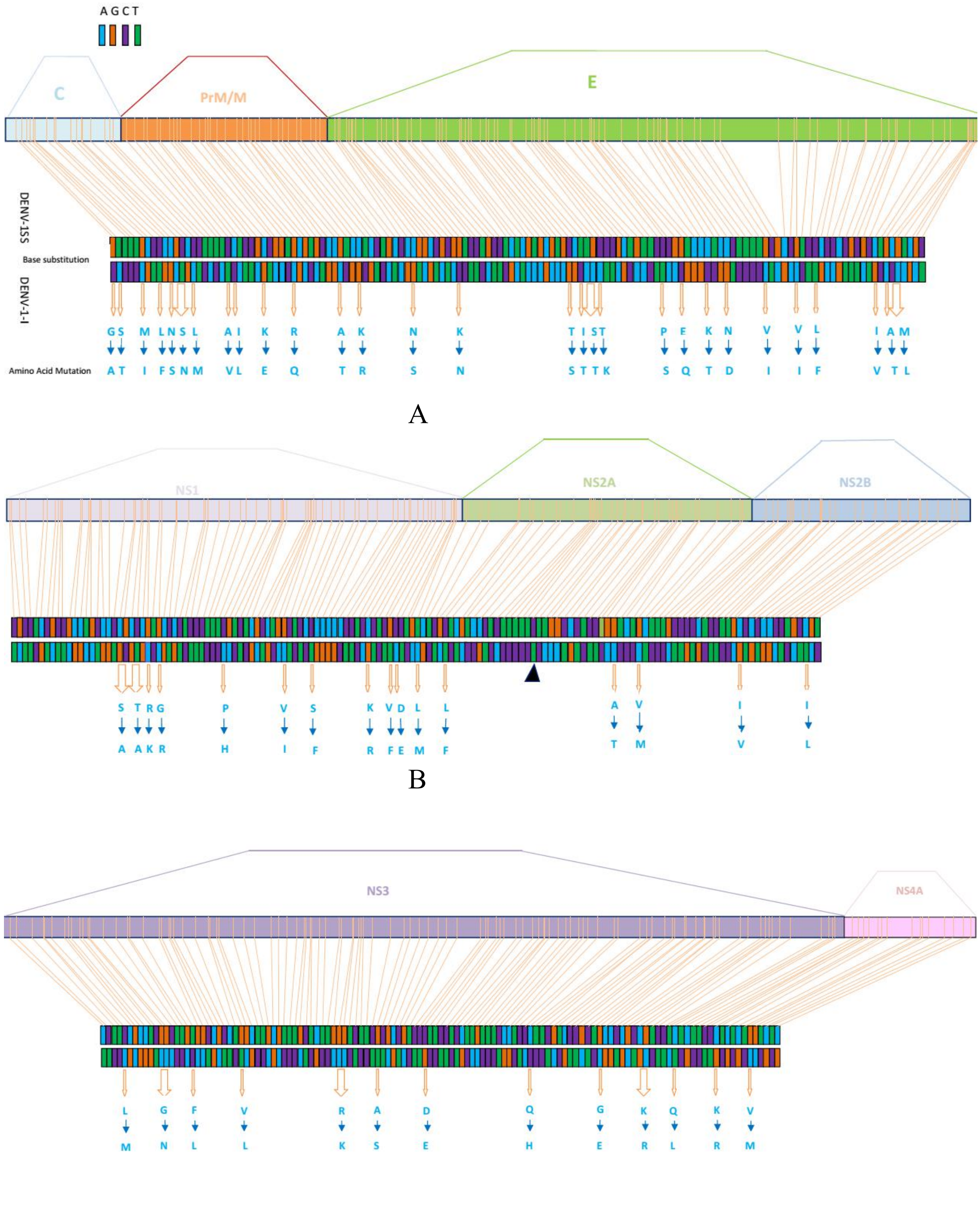

A G C T
C
PrM/M
E
DENV-1SS
Base substitution
DENV-1-I
Amino Acid Mutation
A
NS1
NS2A
NS2B
B
NS3
NS4A
C

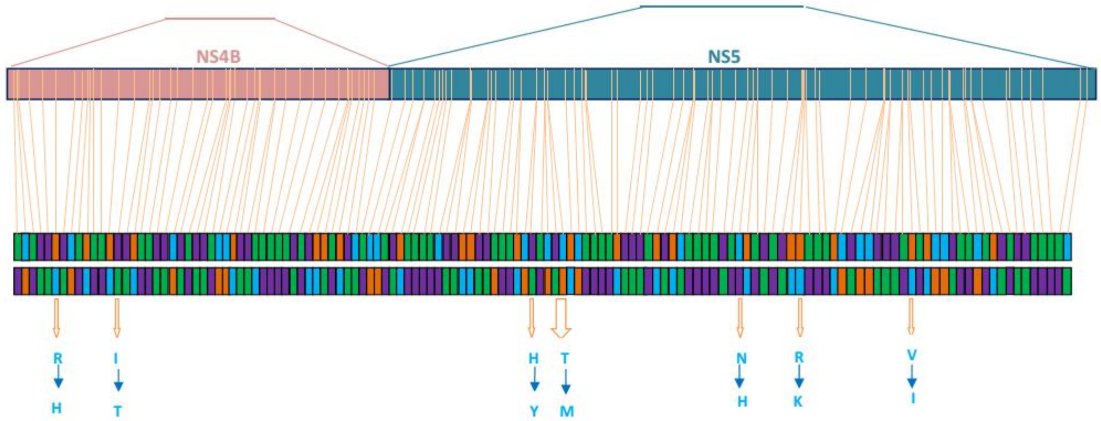

D

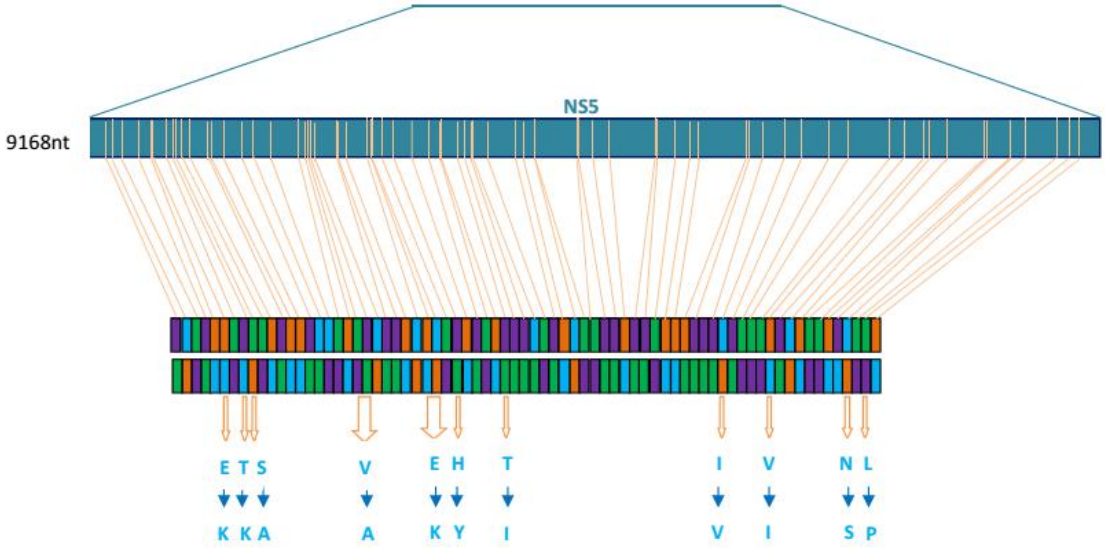

E

Supplementary Figure 1. Base substitutions and amino acid mutations of DENV-1 local isolate strains (DENV-1-I) in 2017 compared to the DENV-1 standard strain (DENV-1SS) (Hawaii,1944). ▲ Indicates that the mutation only existed in the YN17741 strain
