# Supplementary material for: Complete Genome Characterization of the 2017 Dengue Outbreak in Xishuangbanna, a Border City of China, Burma and Laos
Source: Front Cell Infect Microbiol. 2018 May 8;8:148. doi: 10.3389/fcimb.2018.00148 (PMC5951998; doi:10.3389/fcimb.2018.00148)
Supplement: Supplementary file 4 [file Image_2.PDF]

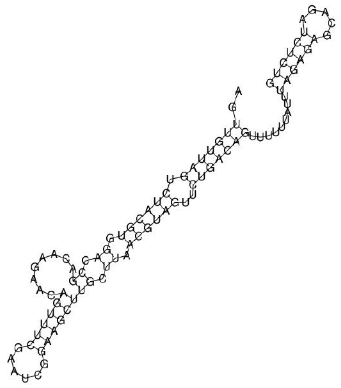

A

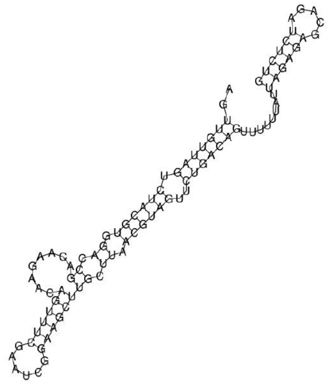

B

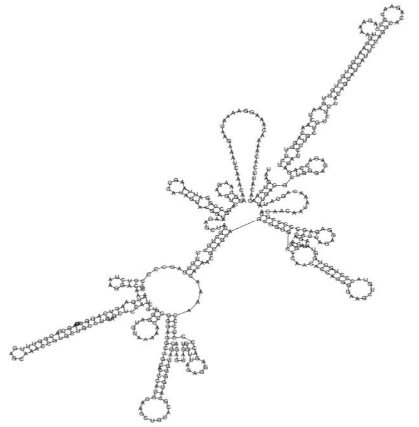

C

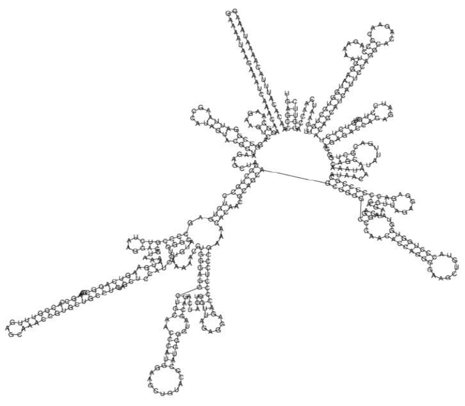

D

Supplementary Figure 2. RNA secondary structure prediction based on the untranslated regions of the standard and isolated strains. (A and C) RNA secondary structure of the 5’ (A) and 3’UTR (C) of the standardstrain. (B and D) RNA secondary structure of the 5’UTR (B) and 3’UTR (D) of the local isolates.
