# Supplementary material for: Complete Genome Characterization of the 2017 Dengue Outbreak in Xishuangbanna, a Border City of China, Burma and Laos
Source: Front Cell Infect Microbiol. 2018 May 8;8:148. doi: 10.3389/fcimb.2018.00148 (PMC5951998; doi:10.3389/fcimb.2018.00148)
Supplement: Supplementary file 5 [file Image_3.PDF]

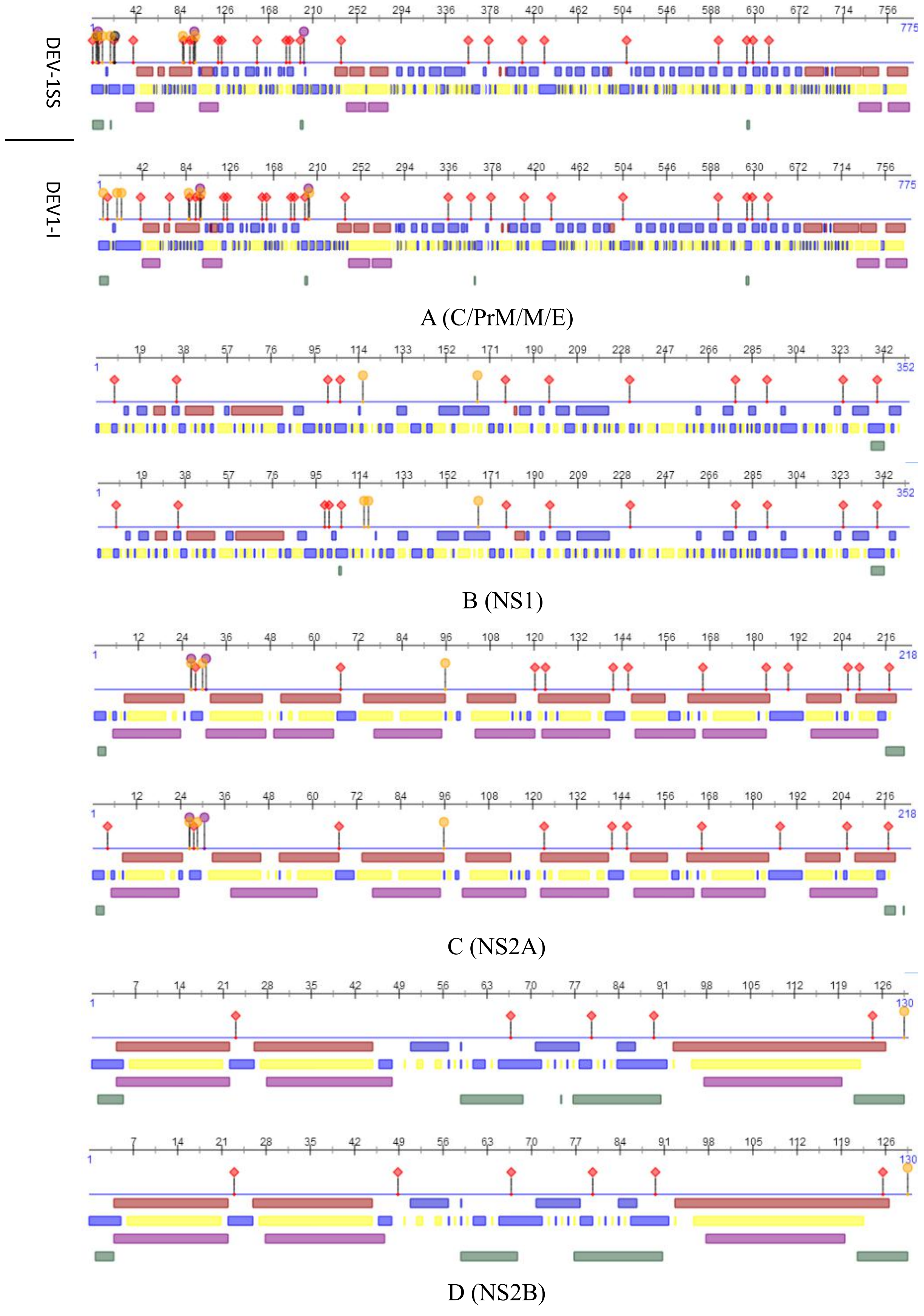

DEV-1SS
DEV1-I
A (C/PrM/M/E)
B (NS1)
C (NS2A)
D (NS2B)

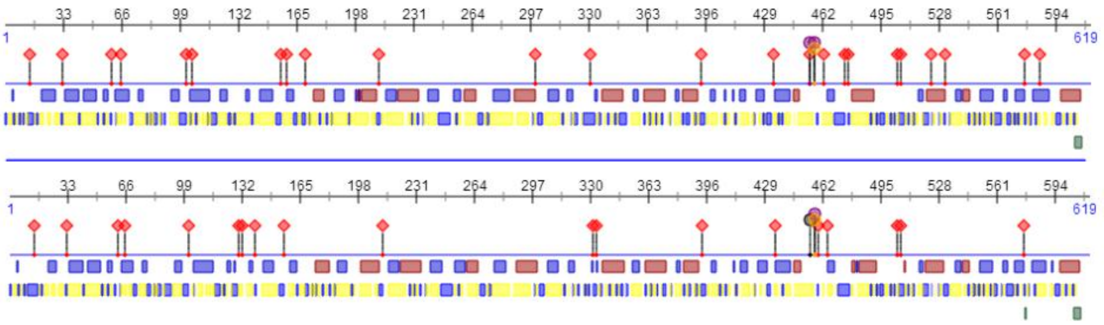

E (NS3)

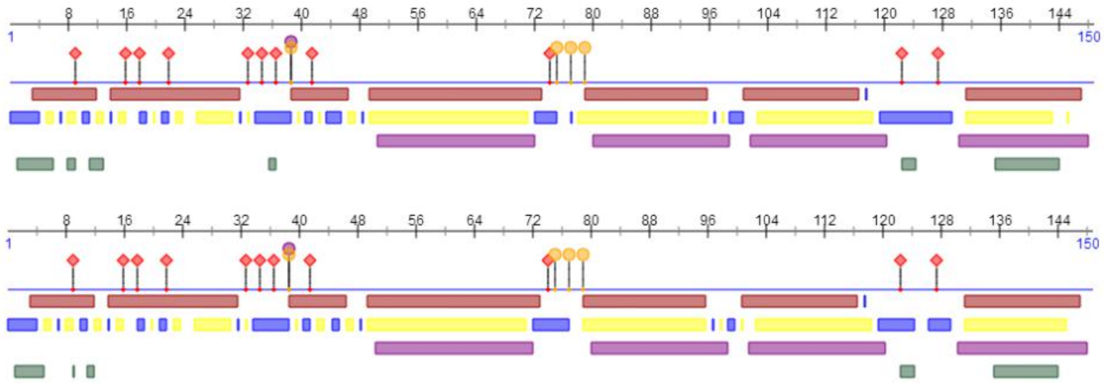

F (NS4A)

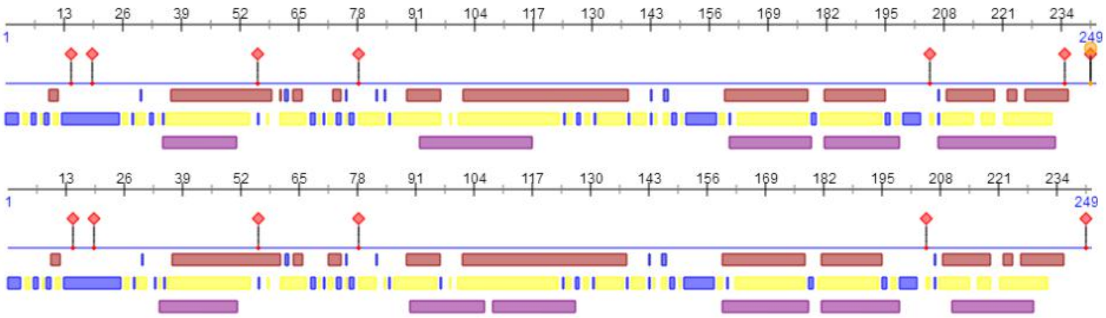

G (NS4B)

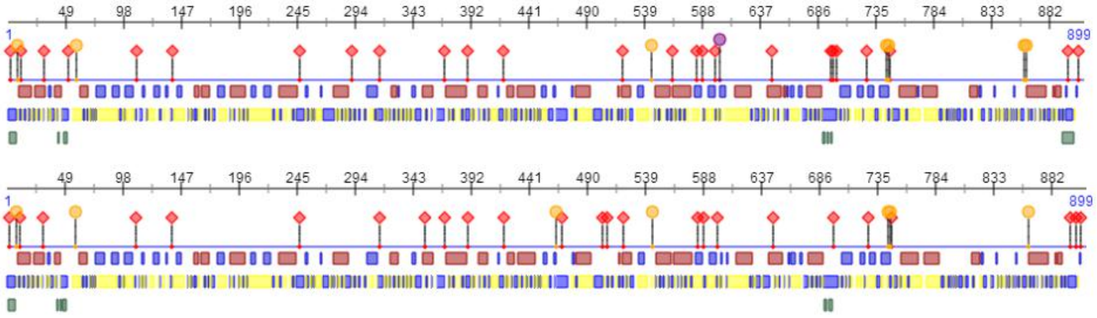

H (NS5)

Supplementary Figure 3. Secondary structure prediction of the CDS for the standard strain (DENV-1SS) and local isolate strains (DENV-1-I). The black dot indicates the nucleotide-binding region; the purple dot indicates the RNA-binding region; the yellow dot indicates the DNA-binding region; and the red rhombus indicates the protein-binding region. Blue and red in the first line denote the strand and helix regions, respectively. Blue and yellow in the second line denote the exposed and

buried regions, respectively. Purple in the third line refers to the helical transmembrane regions, and green in the fourth line denotes the disordered regions.
